# Plant and animal-derived fusion nanovesicles rescue inflammation-compromised osteogenic potential of periodontal ligament stem cells

**DOI:** 10.3389/fcell.2025.1512238

**Published:** 2025-02-27

**Authors:** Jingxiong Lin, Manchun Li, Linglu Wang, Xingyu Lu, Quanle Xu, Hongbo Chen, Dongling Dai

**Affiliations:** ^1^ School of Pharmaceutical Sciences (Shenzhen), Shenzhen Campus of Sun Yat-sen University, Shenzhen, Guangdong, China; ^2^ College of Life Sciences, Northwest A&F University, Xianyang, Shaanxi, China; ^3^ Endoscopy Center and Gastroenterology Department, Key Laboratory for Precision Diagnosis and Treatment of Pediatric Digestive System Diseases, Shenzhen Children’s Hospital, Shenzhen, China

**Keywords:** plant-derived nanovesicles, TNFR1, fusion nanovesicles, inflammatory microenvironment, periodontal ligament stem cells, osteogenic differentiation

## Abstract

Periodontitis is a chronic inflammatory disease affecting the supporting tissues of the teeth and has emerged as a global public health issue. Current therapies primarily address pathogenic factors and alleviate symptoms, with limited options available for complete restoration and reconstruction of already absorbed periodontal bone tissue. In this study, we developed a nanotherapeutic strategy utilizing fusion nanovesicles (FVs) to modulate the inflammatory microenvironment and create a regenerative niche for periodontal ligament stem cells (PDLSCs), which play a crucial role in periodontal tissue repair. The FVs are composed of *Scutellaria baicalensis* nanovesicles (SBNVs) with anti-*Porphyromonas gingivalis* (*P. gingivalis*) and anti-inflammatory properties, combined with PDLSC membrane-derived nanovesicles genetically engineered to express TNFR1. These FVs preserved the biological activity of SBNVs and the immunomodulatory function of PDLSCs. Additionally, FVs effectively captured and cleared TNF-α from the microenvironment through TNFR1. Moreover, FVs alleviated the inflammatory response of PDLSCs induced by *P. gingivalis*-LPS (Pg-LPS) and TNF-α, restoring their proliferation, migration, and osteogenic differentiation capabilities. Hence, this nanotherapeutic strategy holds great potential for treating periodontitis.

## 1 Introduction

Periodontitis is a chronic inflammatory disease characterized by the destruction of tooth-supporting tissues, leading to significant impairment of oral health and recognized as one of the most prevalent chronic inflammatory diseases globally. According to the 2019 epidemiological survey, the global number of individuals suffering from severe periodontitis has reached 1.1 billion (Chen et al., 2021). Clinically, periodontitis is characterized by gingival inflammation and bleeding, the formation of periodontal pockets, and the loss of alveolar bone, which can ultimately lead to tooth loss (Deng et al., 2021). Recent studies have established an association between periodontal disease and various systemic diseases, including diabetes (D’Aiuto et al., 2018), cardiovascular diseases (Ye et al., 2022), Alzheimer’s disease (Schwahn et al., 2022), and osteoporosis (Potempa et al., 2017), imposing a significant burden on both society and the economy. Current clinical treatments for periodontitis include basic therapy (such as supragingival cleaning, subgingival debridement, etc., aimed at controlling plaque and removing local pathogenic factors), drug therapy, and surgical interventions (Eberhard et al., 2015). While the use of antibiotics as adjunctive therapy in periodontal disease treatment has shown effectiveness, there is a concern regarding antibiotic resistance (Herrera et al., 2008; Song et al., 2024). Although basic periodontal treatment has achieved certain effectiveness in addressing pathogenic factors and alleviating symptoms, challenges remain in achieving complete restoration and reconstruction of already absorbed periodontal bone tissue (Graziani et al., 2017). Currently, for patients with periodontitis accompanied by bone resorption, guided tissue regeneration (GTR) or bone grafting surgery are commonly employed. However, predicting the outcome of these surgeries can be challenging (Jepsen et al., 2023). Therefore, the treatment of bone loss caused by periodontitis continues to be a complex issue.

Periodontal ligament stem cells (PDLSCs) are multipotent stem cells in the periodontal ligament tissue, which can differentiate into various types of tissue cells, directly participate in the regeneration and repair of periodontal tissues (Iwayama et al., 2022). However, in patients with periodontitis, the regenerative capacity of PDLSCs is severely impaired by the bacterial plaque microenvironment and the host’s immune-inflammatory microenvironment, contributing to the disruption of bone remodeling balance (Algate et al., 2016). Dental plaque biofilm is the initiating factor of periodontal disease(Darveau, 2010). As the key pathogenic bacteria forming plaque biofilm, *Porphyromonas gingivalis* (*P. gingivalis*) contributes to the destruction of periodontal tissues by secreting various toxic factors (How et al., 2016). *Porphyromonas gingivalis*-lipopolysaccharides (Pg-LPS), as bacterial endotoxins, activate signaling pathways such as Toll-like receptors (TLR), ultimately leading to enhancement of host immune inflammatory responses and bone resorption (Shaddox et al., 2013; Guo et al., 2018; Zhang et al., 2018). Therefore, developing safe and effective non-antibiotic treatment strategies to inhibit pathogenic bacteria and reduce inflammation is crucial in the management of periodontitis (Song et al., 2022; Song et al., 2023). Furthermore, as a major pro-inflammatory factors, tumor necrosis factor-alpha (TNF-α) significantly increases in the gingival crevicular fluid of periodontitis patients. It not only promotes bone resorption but also inhibits bone formation, with its expression level closely related to the severity of periodontitis (Tervahartiala et al., 2001; Passoja et al., 2010; Pan et al., 2019). TNF receptor 1 and 2 (TNFR1 and TNFR2) are characteristic members of the TNF receptor superfamily, which are essential for stimulating downstream signaling through their interactions with the ligand TNF-α. Notably, TNFR1 contains a death domain in its cytoplasmic region, which allows it to initiate cell death and inflammation pathways (Lo, 2025). Although anti-TNF-α therapy has been proven beneficial for the treatment of periodontitis, TNF-α monoclonal antibody therapy may lead to autoimmune and skin adverse reactions (Lima et al., 2004; Di Paola et al., 2007; Mayer et al., 2009; Majdi Abunemer et al., 2023). Therefore, further exploration and understanding of the specific mechanisms of targeting TNF-α for inflammation and regeneration of periodontal tissues are necessary.

In recent years, mesenchymal stem cell (MSC) transplantation therapy has emerged as a promising approach for promoting periodontal tissue regeneration, among which the paracrine mechanism involving the secretion of extracellular vesicles (EVs) and proteins is widely recognized as the main mechanism (Hynes et al., 2012; Phan et al., 2018; Nuñez et al., 2019). Therefore, to address the limitations of MSC transplantation therapy, such as the potential for tumorigenesis, limited survival of transplanted cells, and variability in treatment outcomes, there has been growing interest in cell-free therapies using cell membrane-derived vesicles (CMVs) from MSC sources. CMVs are membrane particles secreted or prepared from almost all living cells, characterized by a phospholipid bilayer membrane structure. Based on their origin, CMVs could be classified into naturally occurring extracellular vesicles, which include three subtypes: exosomes (with a size range of 30–150 nm), ectosomes (with a size range of 150–1,000 nm), and apoptotic EVs (with a size range of 100 nm to 5 μm), as well as artificial extracellular vesicles (aEVs) derived from cell membranes, which have a size similar to that of exosomes (Li et al., 2024). Compared to EVs, aEVs, also named as nanovesicles (NVs), offer unique advantages such as higher yield and the ability to confer unique biological characteristics through genetic modification, such as serving as substitutes for neutralizing antibodies (Xu et al., 2020). In addition, plant-derived nanovesicles (PDNVs) are becoming the next-generation of nano-therapeutic drugs, with the advantages of biocompatibility, cost-effectiveness, eco-friendliness, and ease of large-scale production (Chen et al., 2023; Feng et al., 2023; Fang et al., 2024; Chai et al., 2025). Reports have shown that several PDNVs promoted bone formation in osteoporosis through natural lipids, microRNA, or other substances they contain (Zhao et al., 2024a). Therefore, PDNVs have great potential to harness the natural biological activity of their parent plants to treat periodontitis. However, compared to CMVs, PDNVs lack targeting ability (Fang et al., 2024). To address these limitations, we propose a strategy based on multifunctional animal and plant fusion nanovesicles, aiming to reshape the periodontal pathological microenvironment and promote the restoration of the normal function of PDLSCs through the synergistic effects of multiple functions.

**FIGURE 1:**
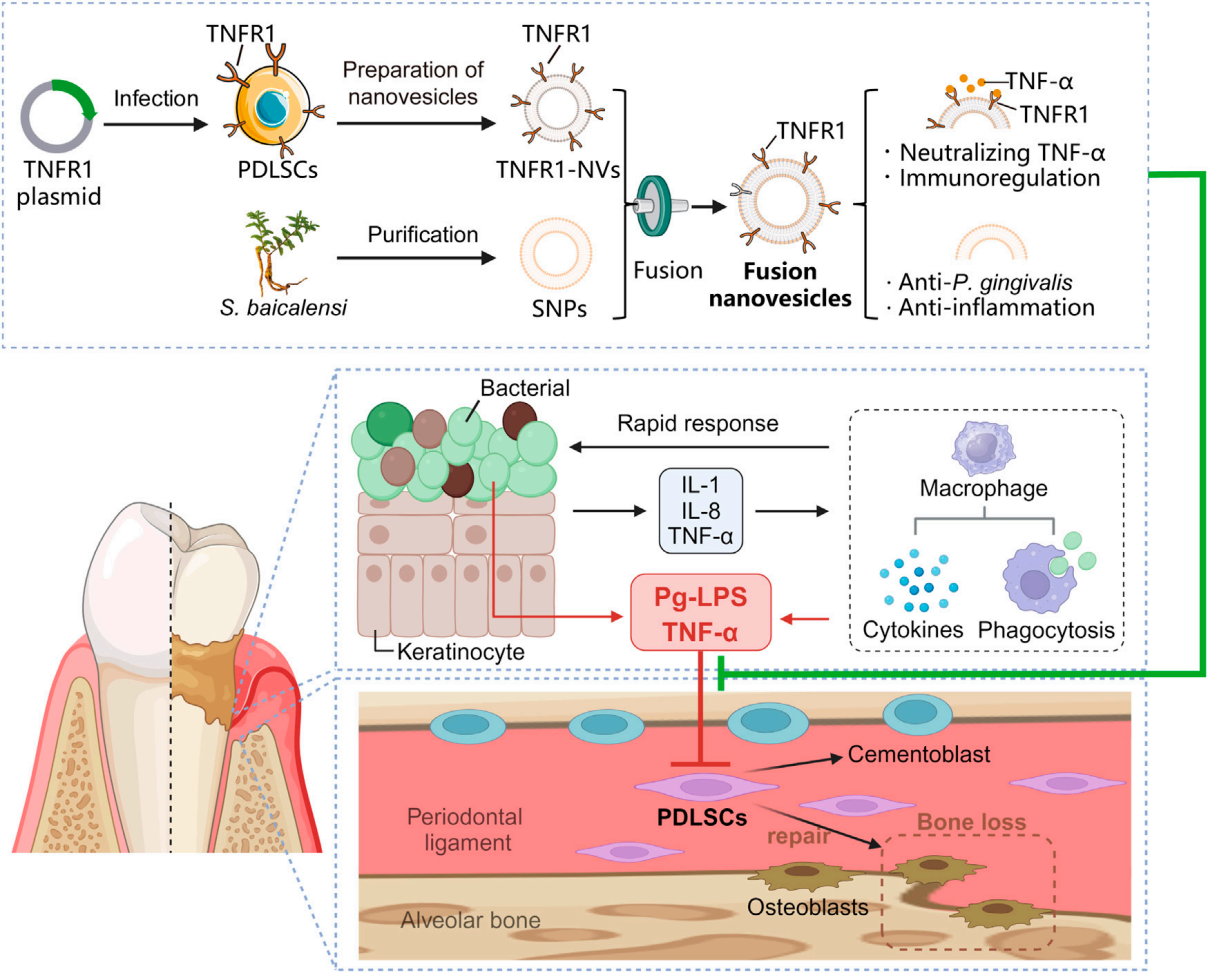
Schematic illustration showing the preparation process of FVs and their therapeutic effects on the osteogenesis of periodontal ligament stem cells.

To address the key issues in treating bone loss associated with chronic periodontitis, it is necessary to modify the periodontal pathological microenvironment and promote the regenerative properties of endogenous pluripotent stem cells. In this study, we developed multifunctional fusion nanovesicles (FVs) by fusing SBNVs with TNFR1-NVs from genetically engineered PDLSCs (Figure 1). We demonstrated that FVs not only maintained the bioactivity of SBNVs and TNFR1-NVs but also exhibited effective immune-regulatory functions. These FVs were capable of reshaping the inflammatory microenvironment induced by Pg-LPS and TNF-α. *In vitro* experiments using PDLSCs inflammation models showed that FVs effectively regulated the inflammation response and osteogenic differentiation of PDLSCs. The findings suggest that our strategy may provide a universal nanocarrier platform targeting the inflammatory microenvironment for regenerative therapy in periodontitis-associated bone loss.

## 2 Methods

### 2.1 Cell culture

HEK293T (human embryonic kidney cell line), RAW267.4 cells (mouse mononuclear macrophage cell line) were cultured in DMEM (Gibco, Cat #C11995500BT) supplemented with 10% FBS (ExCell) and 1% penicillin-streptomycin (EallBio, Cat #03.12001A). Periodontal ligament stem cells (PDLSCs) were cultured in α-MEM (Gibco, Cat #8123188) containing 10% FBS and 1% penicillin-streptomycin. All cells were grown in a 37°C and 5% CO2 incubator.

### 2.2 Isolation of human periodontal ligament stem cells (PDLSCs)

The extracted teeth were immediately placed in cold PBS containing 5% penicillin-streptomycin. On a sterile bench, the tooth crowns were disinfected with 75% ethanol and rinsed repeatedly with PBS. 1/3 Periodontal ligament tissue from the tooth root was scraped off and digested with 3 mg/mL type I collagenase (Sigma-Aldrich, Cat #C0130) in 37°C for 60 min. After digestion, the solution was neutralized with α-MEM containing 10% FBS and then centrifuged. Finally, the PDLSCs were cultured in an incubator at 37°C with 5% CO2.

### 2.3 *In vitro* osteogenic induction in PDLSCs

According to the instructions, the osteogenic induction differentiation kit (Cyagen, Cat #HUXXC-90021) was used to induce PDLSCs for osteogenic differentiation. PDLSCs were seeded at a density of 2 × 10^4^ cells/cm^2^. When the cell confluence reached 70%, the medium was changed to osteogenic induction differentiation medium. The cells were treated for 21 days, with the medium changed every 3 days. Alizarin red staining was used to observe the morphological changes and growth of cells. The kit includes: OriCell^®^ Basal Medium (177 mL; Cat #BLDM-03011), OriCell^®^ Premium Fetal Bovine Serum (20 mL; Cat #FBSSR-01021), and OriCell^®^ Supplement for Osteogenic Differentiation (3 mL; Cat #HUXXC-04021). In addition, 10 μg/mL Pg-LPS (InvivoGen, Cat #tlrl-pglps) or 20 ng/mL TNF-α (NovoProtein, Cat #C008-10 μg) was added to the osteogenic induction medium to simulate the inflammatory microenvironment.

### 2.4 Isolation and purification of PLNVs, CRNVs and SBNVs

Fresh *Pueraria lobata* (for PLNVs), and *Coptidis rhizome* (for CRNVs) and *Scutellaria baicalensis* (for SBNVs) were washed, sliced and then placed into a plant cell wall breaker with pre-chilled PBS solution at a ratio of 1:4 (g/mL) for thorough pulverization. The mixture was then filtered through a 200-mesh sieve to remove remaining unbroken fibrous tissue. The three types of nanovesicles were extracted using the same procedure with the following centrifugation conditions: 1,000 × g for 10 min, 4,000 × g for 20 min, 10,000 × g for 40 min, 40,000 × g for 1 h and 150,000 × g for 2 h. The above centrifugation was performed at 4°C. Finally, the final nanovesicles pellet were resuspended in pre-cooled PBS and further purified using sucrose gradient (8%, 30%, 45%, 60% sucrose in 20 mM Tri-CI, pH 7.2) centrifugation at 150,000 × g for 2 h (Wang et al., 2013; Huang et al., 2023). The nanovesicles from the bands at the 8%/30% and 30%/45% interfaces, where the majority of the nanovesicles were enriched, were collected, suspended in ice-cold PBS, and ultracentrifuged at 150,000 × g for 2 h. The protein concentrations of PLNVs, CRNVs and SBNVs were determined using the BCA assay (Beyotime, Cat #p0009), which served as the basis for dosing.

### 2.5 Preparation of TNFR1-NVs and NVs

To obtain PDLSCs stably expressing TNF receptor 1 (TNFR1), the target plasmid (pCDH-CMV-Homo-TNFRSF1A-EGFP-EF1-puro, IGEbio) was co-transfected with lentiviral packaging and envelope plasmids into HEK-293T cells using Lipofectamine 3000 (ThermoFisher, Cat #L3000001). The supernatant containing lentivirus was harvested at 48 h and 72 h post-transfection. After initial purification at 4,000 × g for 10 min, the lentiviral particles were further sedimented at 20,000 × g for 90 min. PDLSCs were infected with the lentiviral particles and selected with puromycin (1 μg/mL; ThermoFisher, Cat #A1113802) to obtain target cells stably overexpressing TNFR1 (Xi et al., 2023).

TNFR1-PDLSCs and PDLSCs were lysed with homogenization medium buffer containing 0.25 M sucrose, 1 mM EDTA, 20 mM Hepes-NaOH and protease inhibitor cocktail (Epizyme, Cat #GRF101) at 4°C overnight. The lysate was homogenized 200 times on ice using a glass homogenizer, followed by centrifugation at 5,000 × g, 4°C for 10 min. The supernatant was centrifuged again at 12,000 × g, 4°C for 20 min. The sediment was resuspended in pre-chilled PBS and then extruded sequentially through 0.45 μm and 0.22 μm lipid extruders to obtain the desired cell membranes and ensure sterilizations (Huang et al., 2023). The protein concentration of TNFR1-NVs and NVs were determined using the BCA assay (Beyotime, Cat #p0009), which served as the basis for dosing.

### 2.6 Preparation of fusion nanovesicles

Fusion nanovesicles was prepared as previously described (Huang et al., 2023). To prepare fusion nanovesicles (FVs), SBNVs and TNFR1-NVs were mixed in a 1:1 ratio (particle/mL) and blended using a handheld ultrasonic probe on ice. Subsequently, the mixture was extruded through a 0.22 μm lipid extruder. Following centrifugation, the FVs sediment were resuspended in pre-chilled PBS.

### 2.7 Characterization experiments

Purified nanovesicles were pipetted onto the surface of copper grids and incubated for 5 min Subsequently, 8 μL of 2% uranyl acetate solution was added and incubated for 10 min under dark conditions. Next, nanovesicles on the copper were washed with distilled water and air-dried in the dark. The morphology of the nanovesicles was examined by transmission electron microscopy (JEM-1400, 120 kV). The size distribution and Zeta potential of the nanovesicles were determined by the NanoBrook90Plus PALS instrument (Brookhaven).

### 2.8 Alkaline phosphatase staining

Alkaline phosphatase (ALP) staining was performed on day 7 of PDLSCs osteogenic induction (Li et al., 2023). Cells in each well were washed with PBS and fixed in 4% paraformaldehyde at room temperature for 30 min. Then, ALP staining was carried out utilizing the ALP staining kit (Beyotime, Cat #C3206) following the manufacturer’s instructions.

### 2.9 Alizarin Red staining

Alizarin Red staining was performed on day 21 of PDLSCs osteogenic induction (Li et al., 2023). Cells in each well were washed with PBS and fixed in 4% paraformaldehyde at room temperature for 30 min. Then, staining was carried out utilizing 1% Alizarin Red S (Cyagen, Cat #HUXXC-90021). After staining, excess dye was washed away with PBS, and the staining levels were observed using a microscope.

### 2.10 RNA isolation and quantitative real-time PCR

Total RNA was extracted from cells using Trizol reagent (Takara Biotech Cat #9108). MiRNA from nanovesicles was extracted using the MiPure Cell/Tissue miRNA Kit (Vazyme, Cat #RC201). Subsequently, RNA concentration was measured by Nanodrop One (Thermo Fisher Scientific, United States). Then, 1 μg total RNA was reverse transcribed into cDNA using All-in-One First Strand cDNA Synthesis Kit (TransGen Biotech, Cat #AT321-01), followed by quantitative real-time PCR (qPCR) using 2×SYBR Green qPCR SuperMix (TransGen Biotech, Cat #AQ601-01-V2) with LightCycler^®^ 96 (Roche). Specific reverse transcription of miRNA was carried out using the miRNA 1st Strand cDNA Synthesis Kit (Vazyme, Cat #MR201-01). All processes were carried out with the manufacturer’s instruction. Fold change of relative gene expression was calculated using the 2^−ΔΔCt^ method normalized to the controls U6 (for miRNA) or β-actin (for mRNA). Primer sequences used in this study are listed in Supplementary Table S1.

### 2.11 Western blot

Total protein was extracted from cells on ice using RIPA lysis buffer (Beyotime, Cat #P0013B) containing protease inhibitor (Epizyme, Cat #GRF101) and protein phosphatase inhibitor (Solarbio, Cat #P1260), and protein concentration was determined by BCA Protein Assay Kit (Beyotime, Cat #P0009). Subsequently, proteins were separated by 10% SDS-PAGE and transferred to PVDF membranes (Millipore). After blocking with TBST containing 5% fat-free dry milk at room temperature for 2 h, the membranes were then separately incubated with the primary antibodies against β-actin (1:5000, Abkine, Cat #ABL1010), GAPDH (1:5000, ZENBIO, Cat #200306-7E4), ALP (1:5000, Abmart, Cat #T55421S), RUNX2 (1:1000, ABclonal, Cat #A2851), IL-1β (1:1000, Servicebio, Cat #GB11113-100), TNF-α (1:500, Servicebio, Cat #GB11188-100), STAT3 (1:1000, CST, Cat #12640S), p-STAT3 (1:2000, CST, Cat #9145S), GFP (1:5000, ABclonal, Cat #AE012) at 4°C overnight. Next, following washing with TBST, the membranes were incubated with HRP-conjugated secondary antibodies (Anti-rabbit IgG, 1:5000, CST, Cat#7074; Anti-mouse IgG, 1:5000, CST, Cat#7076) for 1.5 h. Finally, membranes were washed again with TBST and the protein levels were detected using an enhanced chemiluminescence (ECL) kit (Protein Tech, Cat #PK10003).

### 2.12 Enzyme-linked immunosorbent assay (ELISA)

MSC-NVs and TNFR1-NVs were separately co-incubated with 1 ng/mL TNF-α cytokine in a cell culture incubator for 24 h. Subsequently, centrifugation was carried out at 12,000 × g for 20 min to remove precipitated NVs and TNF-α cytokines bound to NVs. Finally, ELISA assay kits (DAKEWE, Cat #1117202) were employed to detect unbound TNF-α cytokines in the retained supernatant.

### 2.13 Cell viability assay

The viability of PDLSCs was assessed using the Cell Counting Kit 8 (CCK-8) (APExBIO, Cat #K1018). PDLSCs were seeded in 96-well plates at a density of 3 × 10^3^ cells/well and incubated with 10 μg/mL Pg-LPS or 20 ng/mL TNF-α, along with various drug treatment groups, for 24 h, 48 h, 72 h and 96 h. Next, the medium was replaced with serum-free medium supplemented with 10% CCK-8 and further incubated for 2 h. Subsequently, the absorbance at 450 nm was measured by a microplate reader to determine cell proliferation.

### 2.14 *In vitro* anti-inflammatory effects of different drugs in PDLSCs

PDLSCs were seeded in 6-well plates at a density of 2 × 10^4^ cells/cm^2^ and incubated overnight. To analyze the anti-inflammatory effects of different drugs in PDLSCs stimulated by Pg-LPS or TNF-α, PDLSCs in each well were treated with 10 μg/mL Pg-LPS or 20 ng/mL TNF-α for 6 h. Different drug groups were then added, and after 24 h of incubation, the total RNA and protein of PDLSCs were extracted to analyze the expression levels of inflammatory factors.

### 2.15 ROS level detection

The intracellular levels of reactive oxygen species (ROS) were analyzed using 2′,7′-dichlorodihydrofluorescein diacetate (DCFH-DA, Sigma, Cat #4091-99-0) probe (Zhang et al., 2022). Cells were co-incubated with DCFH-DA (5 μM) at 37°C for 15 min, followed by washing with PBS three times to remove excess DCFH-DA. Cells were then collected and analyzed using flow cytometry, with ROS levels detected in the FITC channel.

### 2.16 Scratch assay

PDLSCs were seeded in 6-well plates at a density of 2 × 10^4^ cells/cm^2^. When cell reached approximately 90%, a straight line was scratched at the bottom of the plate to simulate wound formation. Subsequently, cells were thoroughly washed with PBS to remove cell debris and treated with Pg-LPS, TNF-α, and FVs. The cells were then cultured in serum-free α-MEM. After 12 h, cells that migrated towards the wound line were photographed under a microscope and quantitatively analyzed. Cell migration rate = (Initial scratch area at 0h - Scratch area at 12 h)/Initial scratch area at 0 h × 100.%

### 2.17 Cellular uptake of SBNVs and FVs

For RAW264.7 cell uptake of SBNVs, the SBNVs were stained with 1 μg/mL WGA488 (Biotium, Cat #29022-1) for 15 min. Subsequently, the remaining dye was removed with ultrafiltration 150,000 × g for 30 min. Next, unstained and WGA488-labeled SBNVs (protein concentration: 20 μg/mL) were separately added to RAW264.7 cells seeded on confocal plates. After incubation for 4 h, cells were washed with PBS and fixed with 4% paraformaldehyde. Subsequently, stain with 1 μg/mL DAPI (Beyotime, Cat #C1002) for 15 min at room temperature. Finally, cellular uptake results were observed using a confocal microscope (Zeiss, LSM880) with the following settings: Laser: 488 nm (5.0%); Detector Gain: 540.0; 63× oil immersion objective.

For PDLSCs uptake of FVs, SBNVs were stained with 1 μg/mL WGA594 (Biotium, Cat #29023-1) for 15 min and then fused with GFP-tagged TNFR1-NVs to obtain two fluorescently labeled FVs. Subsequently, the remaining dye was removed with ultrafiltration 150,000 × g for 30 min. Next, FVs (protein concentration: 20 μg/mL) were added to PDLSCs seeded on confocal plates. After incubation for 4 h, the cells were washed with PBS and fixed with 4% paraformaldehyde. Subsequently, stain with 1 μg/mL DAPI (Beyotime, Cat #C1002) for 15 min at room temperature. Finally, the cellular uptake results were observed using a confocal microscope (Zeiss, LSM880) with the following settings: Lasers, 488 nm (5.0%); Lasers, 561 nm (6.5%); Detector Gain: 540.0; 63× oil immersion objective.

### 2.18 *In vitro* experiments of miR-21-5p mimics

Using miR-21-5p mimics to investigate its effects on Pg-LPS and TNF-α-induced PDLSCs, with a sense strand sequence of 5′-UAG​CUU​AUC​AGA​CUG​AUG​UUG​A-3′. Both the miR-21-5p mimics and scrambled controls were synthesized by Suzhou Genepharma. Transfection was conducted using siRNA-Mate™ transfection reagent (GenePharma, Cat #G04008) following the operation manual. After transfection, osteogenic induction culture was conducted on PDLSCs.

### 2.19 miRNA sequencing

Total RNA was extracted using Trizol kit (Invitrogen, Carlsbad, CA, USA). After constructing the cDNA library, sequencing was performed using Illumina Illumina HiSeq Xten by Gene *Denovo* Biotechnology Co. (Guangzhou, China). All of the clean tags were aligned with small RNAs in GeneBank database(Release 209.0) and Rfam database(Release11.0) to remove rRNA, scRNA, snoRNA, snRNA and tRNA. Subsequently, the clean tags were searched against miRBase database (Release 22) to identify known (Species studied) miRNAs. The miRNA expression level was calculated and normalized to transcripts per million (TPM).

### 2.20 Detection of plant lipids

Plant lipids contents were detected by MetWare (https://www.metware.cn/) based on the AB Sciex QTRAP 6500 LC-MS/MS platform. The sample stored at −80°C refrigerator was thawed on ice. A 200 μL normal saline was added into the sample and vortexed for 3 min. Mix the sample and 1 mL of the extraction solvent (MTBE: MeOH = 3:1, v/v) containing internal standard mixture. After whirling the mixture for 15 min, 200 μL of ultrapure water was added. Vortex for 1 min and centrifuge at 12,000 × g for 10 m500 μL of the upper organic layer was collected and evaporated using a vacuum concentrator. The dry extract was reconstituted using 200 μL mobile phase B prior to LC-MS/MS analysis. The sample extracts were analyzed using an LC-ESI-MS/MS system (UPLC, ExionLC AD, https://sciex.com.cn/; MS, QTRAP^®^ 6500+ System, https://sciex.com/). The analytical conditions were as follows, UPLC: column, Thermo Accucore™C30 (2.6 μm, 2.1 mm × 100 mm i.d.); solvent system, A: acetonitrile/water (60/40,V/V, 0.1% formic acid, 10 mmol/L ammonium formate), B: acetonitrile/isopropanol (10/90 V/V, 0.1% formic acid, 10 mmol/L ammonium formate); gradient program, A/B (80:20, V/V at 0 min, 70:30 V/V at 2.0 min, 40:60 V/V at 4 min, 15:85 V/V at 9 min, 10:90 V/V at 14 min, 5:95 V/V at 15.5 min, 5:95 V/V at 17.3 min, 80:20 V/V at 17.3 min, 80:20 V/V at 20 min; flow rate, 0.35 mL/min; temperature, 45°C; injection volume: 2 μL. The effluent was alternatively connected to an ESI-triple quadrupole-linear ion trap (QTRAP)-MS.

### 2.21 Statistical analysis

All data were analyzed using GraphPad Prism Ver 9.5.1, and presented as mean ± standard deviation (SD). Statistical significance between two groups was analyzed using unpaired two-tailed Student’s t-test (*p < 0.05, **p < 0.01, and ***p < 0.001). For comparisons among multiple groups, one-way analysis of variance (ANOVA) followed by *post hoc* tests was conducted.

## 3 Results

### 3.1 *Scutellaria baicalensis* nanovesicles have anti-*Porphyromonas gingivalis* and anti-inflammatory effects

**FIGURE 2:**
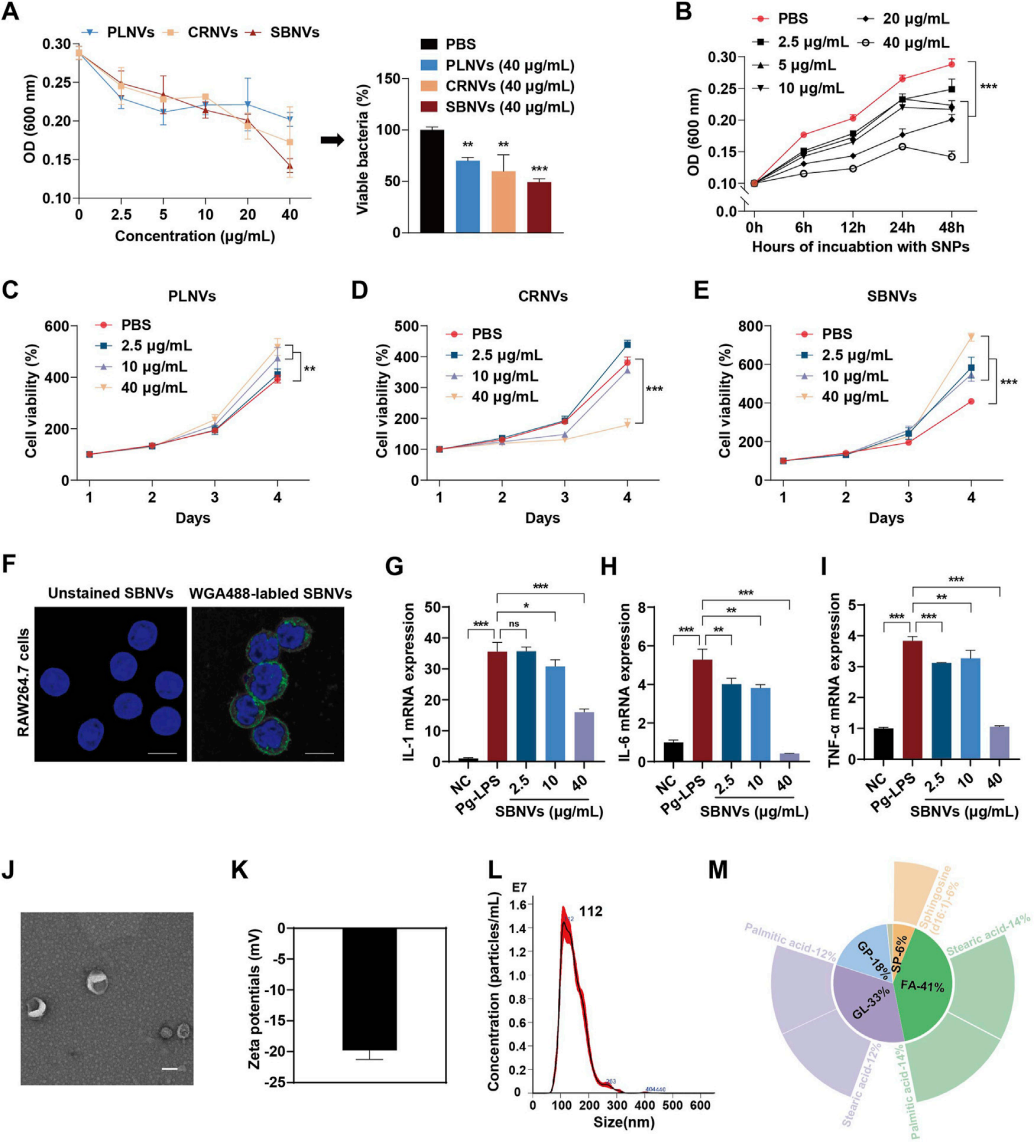
SBNVs have anti-*P. gingivalis* and anti-inflammatory effects. **(A)** *P. gingivalis* were incubated with PLNVs, CRNVs and SBNVs for 48 h. The growth of *P. gingivalis* was quantified by measuring optical density at 600 nm (n = 3). **(B)** *P. gingivalis* were treated with SBNVs for the indicated times. The growth of *P. gingivalis* was quantified by measuring optical density at 600 nm (n = 3). **(C–E)** Cell viability curve of PDLSCs treated with different concentrations of PLNVs, CRNVs and SBNVs respectively for 1–4 days (n = 3). **(F)** Confocal images showing the uptake of SBNVs in RAW264.7 cells. WGA488 was used to label SBNVs (green), and DAPI was used to label cell nuclei. Scale bar: 10 μm. **(G–I)** Relative mRNA expression of IL-1β, IL-6 and TNF-α in Pg-LPS-induced RAW264.7 cells after different treatments (n = 3). Cells in the NC group were treated with medium alone. **(J–L)** Representative TEM image, zeta potential, size distribution of SBNVs. Scale bar: 100 nm. **(M)** The relative content of lipids composition in SBNVs, and the top five secondary lipids with the highest content were displayed on the periphery. ns, no significant difference; *p < 0.05; **p < 0.01; ***p < 0.001.

In order to identify potential PDNVs for the treatment of chronic periodontitis, we first screened medicinal plants mentioned in the literature known for their anti-gingipain activity and anti-inflammatory effects. Five promising medicinal plants were identified for further investigation (Supplementary Figure S1A). *In vitro* antibacterial tests revealed that *Pueraria lobata* nanovesicles (PLNVs), *Coptidis rhizome* nanovesicles (CRNVs), and *Scutellaria baicalensis* nanovesicles (SBNVs) could all inhibit the growth of *Porphyromonas gingivalis* (*P. gingivalis*) in a dose-dependent manner, with SBNVs demonstrating the most significant inhibitory effect (Figure 2A). Moreover, SBNVs exhibited time-dependent inhibition of *P. gingivalis* growth (Figure 2B). The cytotoxicity of these nanovesicles on periodontal ligament stem cells (PDLSCs) was evaluated using a CCK-8 assay. CRNVs inhibited PDLSC proliferation at effective antibacterial concentrations. Conversely, both PLNVs and SBNVs could promote the proliferation of PDLSCs with lower cytotoxicity (Figures 2C–E). Therefore, SBNVs were selected for further investigation. Next, we explored the regulatory ability of SBNVs on the inflammatory response of macrophages, which helps regulate the excessive activation of host immune responses. Confocal images showed that SBNVs could be effectively taken up and internalized in Raw264.7 cells (Figure 2F). Treatment with 40 μg/mL SBNVs significantly reduced the mRNA expression levels of the pro-inflammatory factors IL-1β, IL-6, and TNF-α (Figures 2G–I), and decreased ROS levels in Raw264.7 cells (Supplementary Figures S1B, C). In addition, SBNVs inhibited the expression levels of the M1 macrophage marker protein CD86 in a dose-dependent manner (Supplementary Figures S1D, E). We then characterized the SBNVs using transmission electron microscopy (TEM), zeta potential measurement, and dynamic light scattering (PALS). SBNVs exhibited a typical membrane structure similar to mammalian-derived small extracellular vesicles (sEVs) (Figure 2J), with a zeta potential of approximately −20 mV and an average diameter of approximately 146.3 nm (Figures 2K, L). Subsequent lipidomics analysis of SBNVs revealed a rich content of fatty acids (FA), with sphinganine being one of the top five lipid components (Figure 2M). Sphinganine is a precursor of ceramide synthesis and has broad physiological effects. Metabolite composition analysis of SBNVs using mass spectrometry (MS) revealed four major active small molecules from *Scutellaria baicalensis*, including Baicalin, Baicalein, Wogonoside, and Wogonin (Supplementary Figure S1F). Next, we verified the efficacy of baicalin against *P. gingivalis*. The results show that the minimum inhibitory concentration (MIC) of baicalin is 32 μg/mL (Supplementary Figure S1G). Based on these results, SBNVs demonstrated anti-*P. gingivalis* activity and anti-inflammatory properties, making them as a promising candidate for treating periodontitis.

### 3.2 SBNVs alleviated the inflammatory response and restored the osteogenic differentiation capacity of PDLSCs induced by Pg-LPS

**FIGURE 3:**
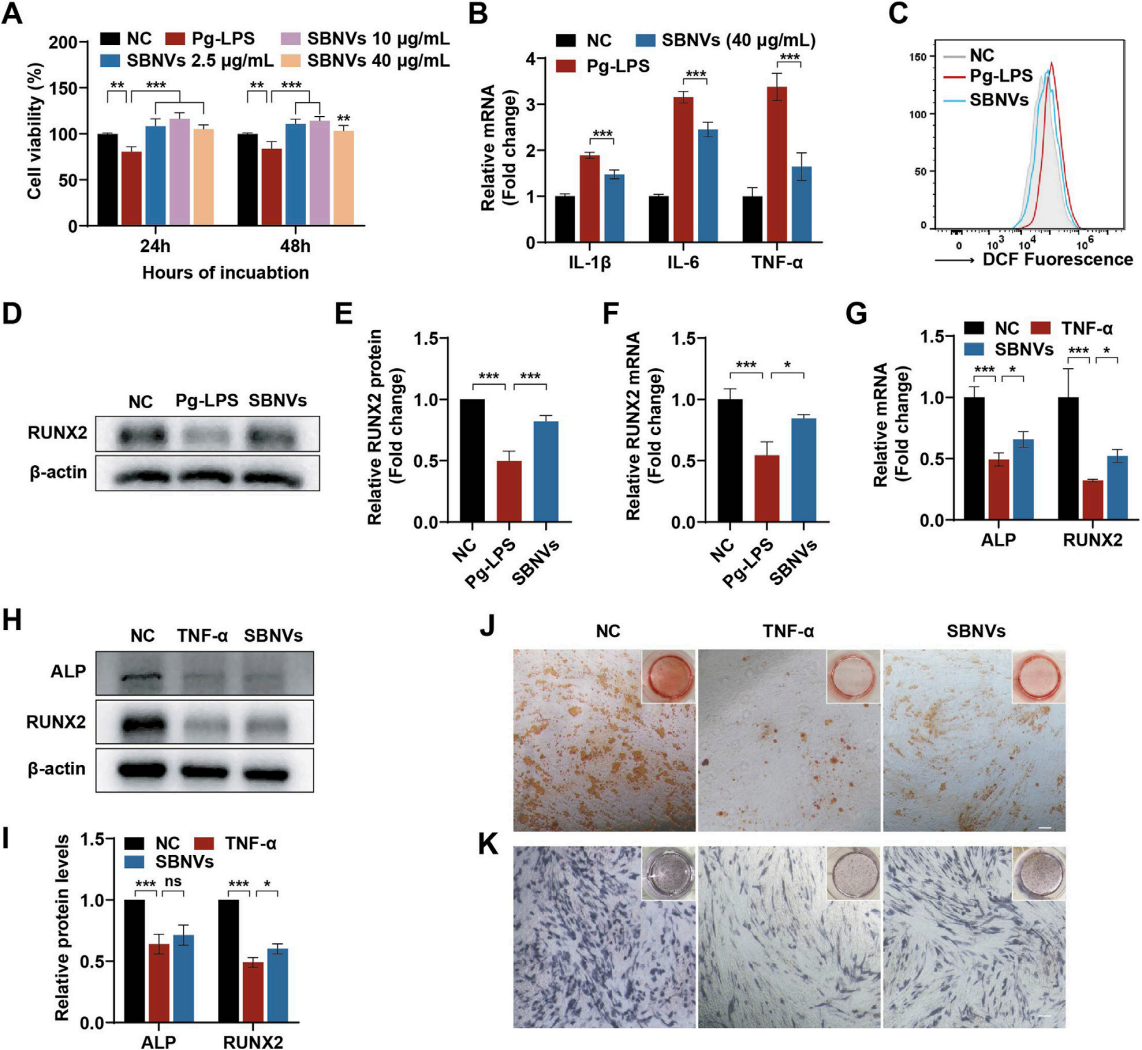
Effects of SBNVs on LPS or TNF-α-induced inflammatory response and osteogenic differentiation. **(A)** The effects of SBNVs on proliferation of Pg-LPS-induced PDLSCs (n = 3). **(B)** Relative mRNA expression of IL-1β, IL-6 and TNF-α in Pg-LPS-induced PDLSCs after SBNVs treatments (n = 3). **(C)** Flow cytometric analysis of intracellular ROS levels in PDLSCs. NC, cells without Pg-LPS treatment. **(D, E)** Typical Western blot bands and quantitative analysis of relative RUNX2 protein levels in Pg-LPS-stimulated PDLSCs at day 7 after SBNVs treatments. **(F)** Relative mRNA expression of RUNX2 in Pg-LPS-stimulated PDLSCs at day 7 after SBNVs treatments (n = 3). **(G)** Relative mRNA expression of RUNX2 in TNF-α-stimulated PDLSCs at day 7 after SBNVs treatments (n = 3). **(H, I)** Typical Western blot bands and quantitative analysis of relative protein levels of ALP and RUNX2 in TNF-α-stimulated PDLSCs at day 7 after SBNVs treatments. **(J, K)** Alizarin Red **(J)** and ALP **(K)** staining shows mineralized nodules of TNF-α-stimulated PDLSCs at days 7 and 21 after SBNVs treatments, respectively. Scale bars, 200 µm. For Panels **(A–C)**, cells in the NC group were treated with medium alone. For Panels **(D–K)**, cells in the NC group were treated with osteogenic differentiation medium alone. *p < 0.05; ***p < 0.001.

It has been reported that factors produced by plaque biofilms and the immune-inflammatory status of the periodontal pathological microenvironment have a negative impact on the biological functions of PDLSCs (Chen et al., 2013). Therefore, evaluating SBNVs under pathological conditions could help elucidate their therapeutic potential for PDLSCs. Given the pathogenic role of *P. gingivalis* in periodontal diseases, Pg-LPS were used to simulate the inflammatory state during *P. gingivalis* infection. First, we investigated the effects of SBNVs on the proliferative capability of PDLSCs in the presence of Pg-LPS. Pg-LPS stimulation significantly reduced the viability of PDLSCs. However, compared to the Pg-LPS group, treatment with various doses of SBNVs effectively promoted the viability of PDLSCs under the influence of Pg-LPS (Figure 3A). Therefore, considering the previous effective dose of SBNVs in the anti-inflammatory effect of macrophages, we decided to select a concentration of 40 μg/mL for SBNVs for subsequent experimental studies. We then investigated the anti-inflammatory and anti-oxidative effects of SBNVs in PDLSCs in an inflammatory environment induced by Pg-LPS. Compared to cells cultured in medium alone, Pg-LPS stimulation significantly increased the mRNA levels of pro-inflammatory mediators IL-1β, IL-6, and TNF-α in PDLSCs (Figure 3B). In contrast, treatment with SBNVs significantly reduced the mRNA expression of these pro-inflammatory mediators and decreased levels of reactive oxygen species (ROS) in PDLSCs (Figure 3C). Furthermore, we investigated the impact of SBNVs on the osteogenic differentiation of PDLSCs in the Pg-LPS inflammatory environment. Compared to the normal control group, Pg-LPS-stimulated PDLSCs showed a significant decrease in the mRNA level of RUNX2, a transcription factor essential for bone formation and osteoblast differentiation, while SBNVs treatment significantly increased the mRNA level of RUNX2 in PDLSCs (Figure 3F). Consistently, Western blot analysis revealed that treatment with SBNVs increased the protein levels of RUNX2 in PDLSCs at day 7 (Figures 3D, E). These results confirmed that SBNVs can enhance the osteogenic differentiation ability of PDLSCs in an inflammatory environment induced by Pg-LPS.

### 3.3 SBNVs had limited effect on alleviating TNF-α-induced inhibition of osteogenic differentiation of PDLSCs

While Pg-LPS has been widely used to simulate the periodontitis microenvironment, a single Pg-LPS model may not fully replicate its complexity. In order to further investigate the biological effects of immune inflammation on PDLSCs, we utilized TNF-α, a major endogenous pro-inflammatory factor, to simulate the host immune inflammatory microenvironment *in vitro*, and then studied the role of SBNVs in regulating the osteogenic differentiation process of PDLSCs. Compared to the normal control group, the mRNA and protein levels of ALP and RUNX2 in PDLSCs were significantly reduced after TNF-α stimulation (Figures 3G–I). Although treatment with SBNVs led to a slight increase in the mRNA and protein expression of ALP and RUNX2, the overall increase was not significant. Further ARS and ALP staining results showed that SBNVs had limited effects in treating TNF-α-induced inhibition of osteogenic differentiation of PDLSCs (Figures 3J, K). These results suggested that additional strategies were needed to enhance the function of SBNVs in response to the destructive effects of TNF-α, which is abundant in periodontitis.

### 3.4 TNFR1-NVs could neutralize TNF-α and promote PDLSCs osteogenic differentiation

**FIGURE 4:**
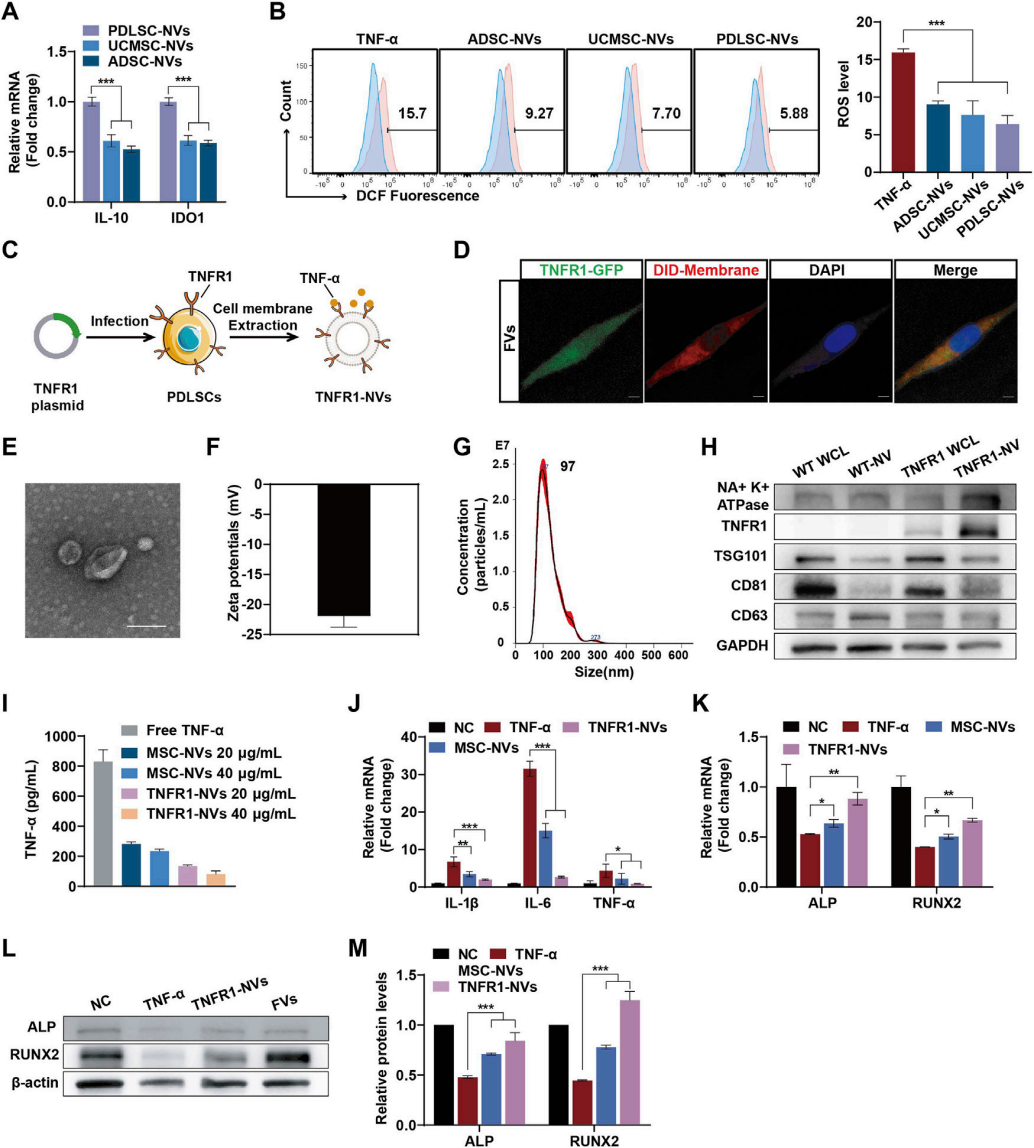
TNFR1-NVs neutralized TNF-α and promoted PDLSCs osteogenic differentiation. **(A)** Relative mRNA expression of IL-10 and IDO1 in NVs from different mesenchymal stem cells (n = 3). **(B)** Flow cytometric analysis of intracellular ROS levels in PDLSCs. The blue portion represents the NC group, which was treated with medium alone. The experimental groups, indicated in red, were treated with TNF-α, either with or without the therapeutic agent. **(C)** Schematic diagram of the preparation of TNFR1-NVs. **(D)** Confocal image showing colocalization of TNFR1-GFP and DID-labeled cell membranes; DAPI was used to label cell nuclei. Scale bar: 10 μm. **(E–G)** Representative TEM image, zeta potential, size distribution of TNFR1-NVs. Scale bar: 100 nm. **(H)** Western blot analysis of the expression of TNFR1-GFP and MSC markers on TNFR1-NVs. WCL, whole cell lysate. **(I)** ELISA assay to detect the ability of NVs to neutralize TNF-α (n = 3). **(J)** Relative mRNA levels of IL-1β, IL-6 and TNF-α in TNF-α-stimulated PDLSCs after different NVs treatment (n = 3). **(K)** Relative mRNA expression of ALP and RUNX2 in TNF-α-stimulated PDLSCs at day 7 after different NVs treatments (n = 3). **(L–M)** Typical Western blot bands and quantitative analysis of relative protein levels of ALP and RUNX2 in TNF-α-stimulated PDLSCs at day 7 after different NVs treatments. For Panelss **(J–M)**, cells in the NC group were treated with osteogenic differentiation medium alone. *p < 0.05; **p < 0.01; ***p < 0.001.

Enhancing the functionality of PDNVs through bioengineering remains a considerable challenge compared to EVs derived from mammalian cells. Studies have shown that nanovesicles (NVs) derived from MSCs have immunomodulatory effects and could be endowed with unique biological properties through physical, chemical, and genetic engineering methods (Xu et al., 2024). Therefore, we proposed the overexpression of TNFR1 on the MSCs through genetic engineering to prepare TNFR1-NVs as decoy receptors for TNF-α, inhibiting its activity. Furthermore, we hypothesized that fusing SBNVs with NVs derived from genetically engineered MSCs would be an efficient strategy to exploit the versatility of SBNVs and the immunosuppressive functions and TNF-α neutralization of NVs, enhancing regulation in response to TNF-α inflammatory environment. To construct TNFR1-NVs, we first compared the immunomodulatory abilities of NVs from three types of MSCs. Compared to umbilical cord mesenchymal stem cells-NVs (UCMSCs-NVs) and adipose-derived mesenchymal stem cells-NVs (ADMSCs-NVs), PDLSC-NVs showed higher mRNA expression levels of immunosuppressive factors IL-10 and IDO (Figure 4A). In addition, PDLSC-NVs significantly reduced the ROS levels in TNF-α-induced PDLSCs (Figure 4B), indicating that PDLSCs were suitable seed cells for functionally targeted modified NVs. The extracted PDLSCs showed a spindle shape and expressed MSC markers CD73 and CD90, while not expressing hematopoietic stem cell markers CD19 and CD45 (Supplementary Figure S2A). *In vitro* experiments of osteogenic and adipogenic differentiation confirmed the multipotency of the extracted PDLSCs (Supplementary Figures S2B, C). PDLSCs overexpressing TNFR1 were established by lentivirus infection (Figure 4C), with TNFR1 expression localized on the cell membrane, as indicated by the co-localization of TNFR1-GFP (green) and DID-labeled cell membrane (red) (Figure 4D). Next, we prepared TNFR1-NVs, and transmission electron microscopy (TEM) and dynamic light scattering (DLS) analysis showed that TNFR1-NVs exhibited a vesicular morphology (Figure 4E), with an average zeta potential of −21 mV (Figure 4F) and an average diameter of 121.9 nm (Figure 4G). In addition, Western blot analysis showed that TNFR1-NVs inherited relevant markers such as TSG101 and CD81, as well as TNFR1-GFP, confirming the successful preparation of NVs derived from PDLSCs overexpressing TNFR1 (Figure 4H).

Next, to validate the potential of TNFR1-NVs neutralizing TNF-α, we co-incubated 1 ng/mL of free TNF-α with different doses of nanovesicles for 24 h, followed by centrifugation to remove the nanovesicles. The remaining unbound TNF-α in the supernatant was detected by ELISA assay. The results showed that while MSC-NVs exhibited a certain degree of non-specific binding ability to TNF-α, TNFR1-NVs showed specific antagonistic function (Figure 4I). We further investigated the anti-inflammatory and osteogenic differentiation effects of TNFR1-NVs on PDLSCs in the TNF-α inflammatory environment. Compared to the control group, treatment with TNF-α significantly increased the mRNA expression of pro-inflammatory mediators IL-1β, IL-6, and TNF-α in PDLSCs. However, after nanovesicle treatment, the mRNA expression of these pro-inflammatory mediators was significantly reduced, with TNFR1-NVs exhibiting stronger anti-inflammatory activity (Figure 4J). Additionally, compared to the control group, the mRNA levels of ALP and RUNX2 (Figure 4K) and protein levels (Figures 4L, M) were significantly increased after TNFR1-NVs treatment. Interestingly, unmodified nanovesicles also showed osteogenic differentiation effects, indicating the immunomodulatory properties of nanovesicles derived from mesenchymal stem cells. The results above indicated that TNFR1-NVs could directly reshape the inflammatory microenvironment induced by TNF-α. Combined with their immunomodulatory property, TNFR1-NVs effectively alleviate the inflammatory response and inhibit TNF-α-induced osteogenic differentiation in PDLSCs.

### 3.5 FVs possessing TNF-α neutralizing and anti-*P. gingivalis* effects promoted the proliferation and migration of PDLSCs in the inflammatory microenvironment induced by Pg-LPS and TNF-α

**FIGURE 5:**
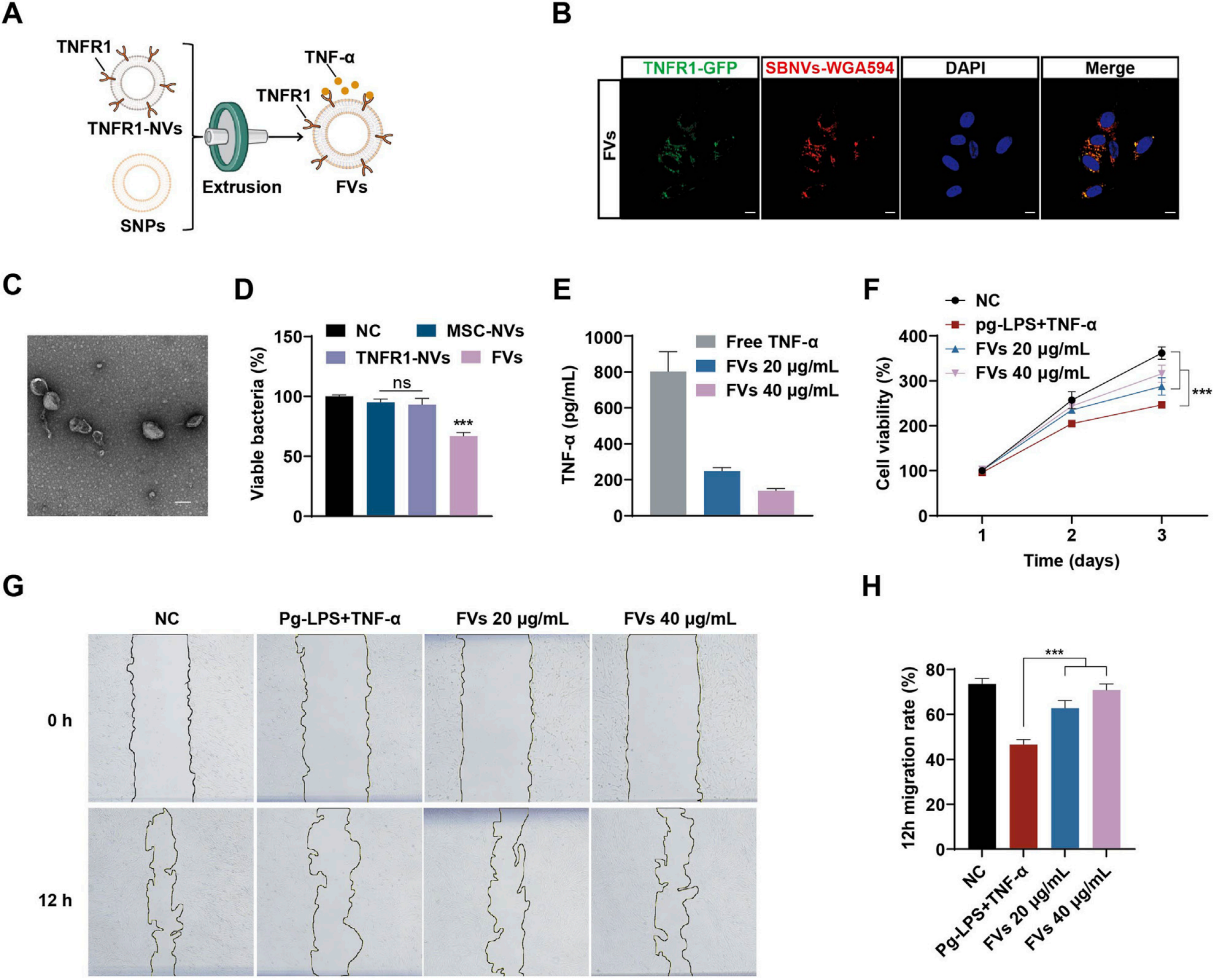
FVs with TNF-α neutralizing and anti-*P. gingivalis* effects promoted PDLSCs proliferation and migration in the inflammatory microenvironment of Pg-LPS and TNF-α. **(A)** Schematic diagram of the preparation of FVs. **(B)** Confocal images showing the uptake of FVs in PDLSCs. WGA594 was used to label SBNVs (green), and DAPI was used to label cell nuclei.Scale bar: 10 μm. **(C)** Representative TEM image of FVs. Scale bar: 100 nm. **(D)** *P. gingivalis* were incubated with different NVs for 48 h. The growth of *P. gingivalis* was quantified by measuring optical density at 600 nm (n = 3). **(E)** ELISA assay to detect the ability of FVs to neutralize TNF-α (n = 3). **(F)** Cell viability curve of the effects of FVs on proliferation of PDLSCs stimulated by Pg-LPS and TNF-α together (n = 3). **(G, H)** Scratch test to determine the effects of FVs on the migration of PDLSCs stimulated by Pg-LPS and TNF-α together (n = 3). In all cases, cells in the NC group were treated with medium alone. ns, no significant difference. ***p < 0.001.

In order to improve targeting and synergistic effects, we fused SBNVs and TNFR1-NVs through membrane extrusion (Figure 5A). Subsequently, we labeled SBNVs with WGA594 and fused them with TNFR1-NVs carrying GFP tags to form FVs to explore their uptake by PDLSCs. Confocal images showed the co-localization of WGA594-labeled SBNVs (red) with TNFR1-NVs (green) around the nuclei of PDLSCs, confirming the successfully preparation of FVs capable of being taken up by PDLSCs (Figure 5B; Supplementary Figure S3A). We further validated whether membrane fusion affected the physical properties of FVs. TEM imaging and DLS analysis showed that FVs exhibited a vesicular morphology (Figure 5C), with an average zeta potential of −20 mV (Supplementary Figure S4A), an average diameter of 142.6 nm (Supplementary Figure S4B). Western blot analysis showed that FVs expressed markers TSG101, CD63, and CD81, as well as TNFR1-GFP (Supplementary Figure S4C). These results indicated that we have prepared engineered plant-animal FVs containing fused SBNVs and TNFR1-NVs. In addition, we investigated the antibacterial and TNF-α neutralizing effects of FVs. While MSC-NVs and TNFR1-NVs had no effect on the growth of *P. gingivalis*, FVs significantly inhibited the growth of *P. gingivalis* with no bactericidal effect (Figure 5D). ELISA assay showed that compared to the control group, FVs could neutralize free TNF-α in a dose-related pattern (Figure 5E). Next, we added 10 μg/mL Pg-LPS and 20 ng/mL TNF-α to the culture medium to create an inflammatory microenvironment. The CCK8 results showed that treatment with different concentrations of FVs effectively promoted the viability of PDLSCs in the inflammatory microenvironment (Figure 5F). Based on the comprehensive analysis of cell viability, we selected 40 μg/mL FVs for subsequent experiments. The scratch assay results showed that treatment with different concentrations of FVs significantly enhanced the migration ability of PDLSCs in the inflammatory microenvironment (Figures 5G, H). In conclusion, the membrane fusion strategy enabled FVs to possess both antibacterial activity against *P. gingivalis* and the ability to neutralize TNF-α.

### 3.6 FVs alleviate the inflammatory response and restore osteogenic differentiation of PDLSCs in the inflammatory microenvironment

**FIGURE 6:**
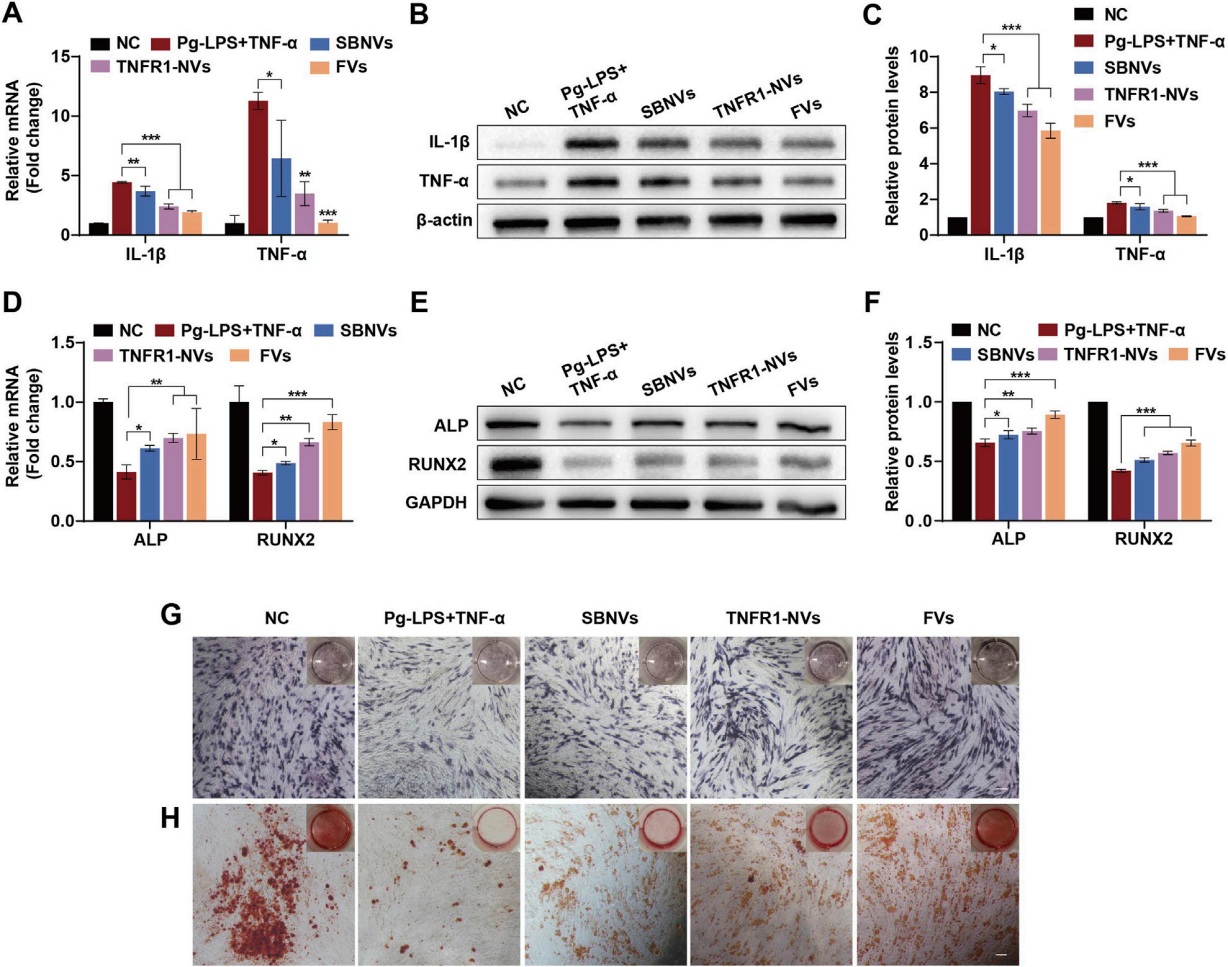
FVs alleviated the inflammatory response and osteogenic differentiation inhibition of PDLSCs in the inflammatory microenvironment. **(A)** Relative mRNA levels of pro-inflammatory cytokines IL-1β and TNF-α in PDLSCs after different treatments (n = 3). **(B, C)** Typical Western blot bands and quantitative analysis of relative protein levels of IL-1β and TNF-α in PDLSCs after different treatments (n = 3). **(D)** Relative mRNA levels of ALP and RUNX2 in PDLSCs at day 7 after different treatments (n = 3). **(E, F)** Typical Western blot bands and quantitative analysis of relative protein levels of ALP and RUNX2 in PDLSCs at day 7 after different treatments (n = 3). **(G, H)** ALP **(G)** and Alizarin Red **(H)** staining shows mineralized nodules of PDLSCs in the inflammatory microenvironment at days 7 and 21 after different treatments, respectively. Scale bars, 200 µm. In all cases, 10 μg/mL Pg-LPS together with 20 ng/mL TNF-α were utilized to simulate the inflammatory microenvironment *in vitro.* For Panels **(A–C)**, cells in the NC group were treated with medium alone. For Panels **(D–H)**, cells in the NC group were treated with osteogenic differentiation medium alone. *p < 0.05; **p < 0.01; ***p < 0.001.

We further investigated the anti-inflammatory and osteogenic effects of FVs on PDLSCs in the inflammatory environment induced by Pg-LPS and TNF-α. Initially, we pre-stimulated PDLSCs with 10 μg/mL Pg-LPS and 20 ng/mL TNF-α for 6 h, followed by adding different treatments for 24 h. Compared to cells treated with medium alone, exposure of PDLSCs to Pg-LPS and TNF-α significantly increased the mRNA levels of the pro-inflammatory mediators IL-1β and TNF-α (Figure 6A). In contrast, treatment with SBNVs, TNFR1-NVs, and FVs significantly decreased the mRNA expression of these mediators, with FVs showing the most pronounced anti-inflammatory effect. Consistent conclusions were drawn from Western blot analysis, showing that FVs reduced the protein levels of IL-1β and TNF-α induced by Pg-LPS and TNF-α in PDLSCs (Figures 6B, C). Compared with the normal control group, exposure of PDLSCs to Pg-LPS and TNF-α showed significantly decreased mRNA levels of ALP and RUNX2 (Figure 6D). However, treatment with SBNVs, TNFR1-NVs, and FVs significantly increased the mRNA levels of ALP and RUNX2 in PDLSCs. Particularly, compared to individual components, FVs exhibited a more significant effect on promoting osteogenic differentiation. Western blot analysis showed similar results, with increased levels of ALP and RUNX2 proteins in PDLSCs after treatments with different interventions (Figures 6E, F). Similarly, ALP staining confirmed FVs effectively reversed the inhibitory effects of Pg-LPS and TNF-α on osteogenic differentiation of PDLSCs (Figure 6G). Furthermore, Alizarin Red staining showed more mineralized nodules for PDLSCs treated with FVs (Figure 6H). These results substantiated that FVs alleviate the inflammatory response and restore the osteogenic differentiation of PDLSCs in the inflammatory microenvironment.

### 3.7 miR-21-5p of fused nanovesicles promoted osteogenic differentiation of PDLSCs by inhibiting STAT3 in the inflammatory microenvironment

**FIGURE 7:**
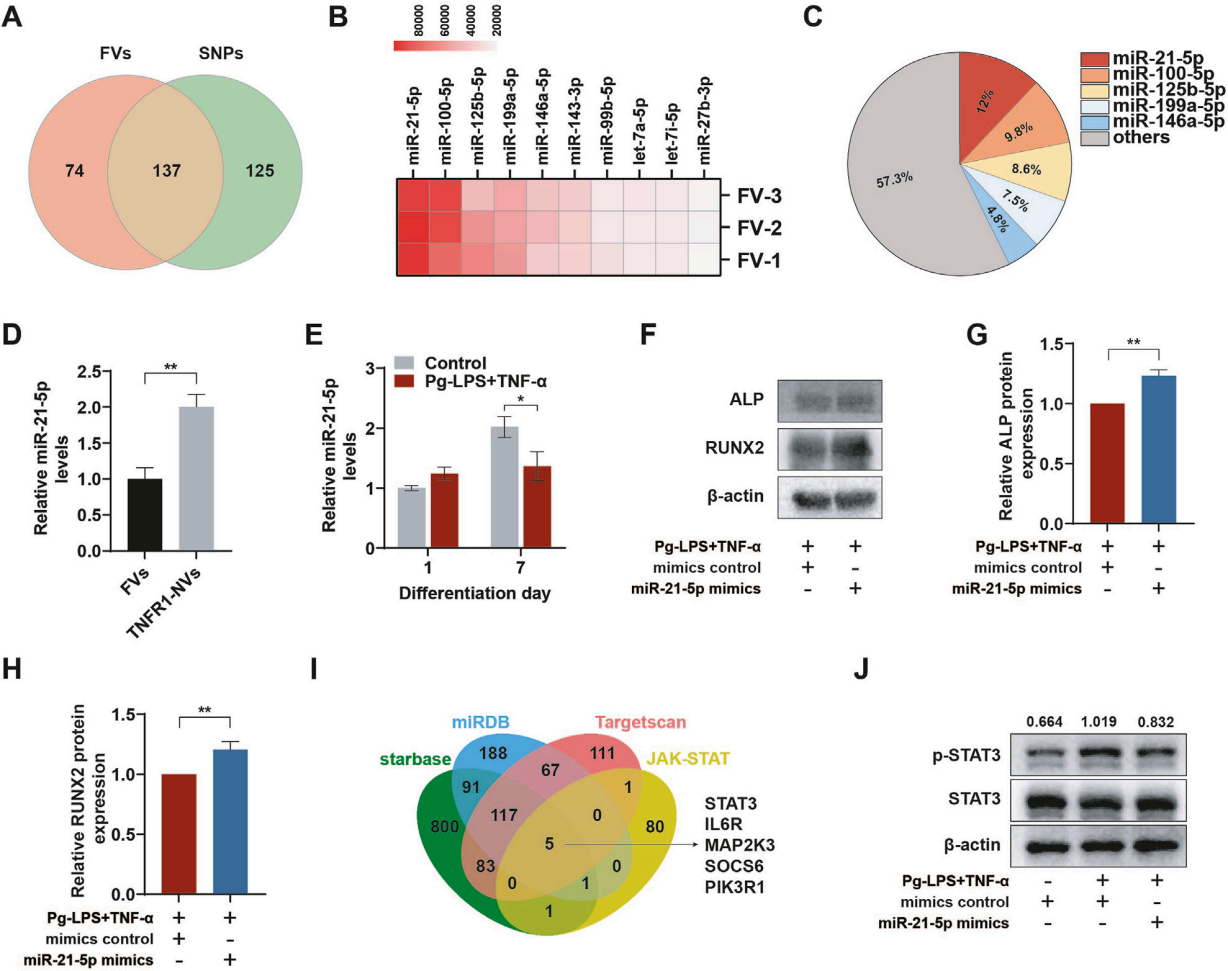
miR-21-5p of fused nanovesicles promoted osteogenic differentiation of PDLSCs by inhibiting STAT3 in the inflammatory microenvironment. **(A)** Venn diagram of miRNAs included in FVs and SBNVs obtained by miRNA-seq. **(B)** Heatmap of the top ten most abundant miRNAs obtained from FVs through miRNA-seq. **(C)** Relative percentage of each miRNA in the total miRNA read counts obtained from FVs through miRNA-seq. **(D)** Relative expression levels of miR-21-5p in FVs and TNFR1-NVs (n = 3). **(E)** The expression level of miR-21-5p was suppressed in the osteogenic differentiation process of PDLSCs in the inflammatory microenvironment (n = 3). **(F–H)** Western blot analysis of the protein levels of ALP and RUNX2 in PDLSCs treated with miR-21-5p mimics or mimics control in the inflammatory microenvironment at day 7 (n = 3). **(I)** Venn diagram showing the overlap between miR-21-5p target genes in three public databases and JAK-STAT pathway genes. **(J)** Western blot analysis of the activation level of STAT3 in PDLSCs overexpressing miR-21-5p at day 7 (n = 3). The values represent the relative ratio of p-STAT3 to total STAT3 for each sample. In all cases, 10 μg/mL Pg-LPS together with 20 ng/mL TNF-α were utilized to simulate the inflammatory microenvironment *in vitro*. *p < 0.05; **p < 0.01.

MiRNA-mediated regulation plays an important role in physiological and pathological processes, providing a new mechanism for intercellular communication.(Xia et al., 2023). We further analyzed the composition of miRNAs in FVs through miRNA-seq. The Venn diagram intersection showed that after membrane fusion, FVs retained the miRNAs from SBNVs and obtained 74 miRNAs from TNFR1-NVs (Figure 7A). Next, based on the total reads of miRNAs in FVs, the top 10 abundant miRNA were sorted (Figure 7B). Among these, the top 5 abundant miRNA in FVs were miR-21-5p, miR-100-5p, miR-125b-5p, miR-199a-5p, and miR-146a-5p, accounting for approximately 42.7% of the total miRNA reads (Figures 7B, C). We further validated the abundant expression of miR-21-5p in TNFR1-NVs and FVs through RT-qPCR (Figure 7D). The content of miR-21-5p in TNFR1-NVs was 2.04 times that in FVs. The difference in the content of miR-21-5p may reflect functional differences between SBNVs and FVs. Next, we determined whether the inflammatory environment affects the expression of miR-21-5p during the differentiation process of PDLSCs into osteoblasts. The results showed that under the stimulation of Pg-LPS and TNF-α, the expression of miR-21-5p increased at day 1, while it was significantly inhibited at day 7 (Figure 7E). Subsequently, we transfected miR-21-5p and control miRNA into PDLSCs, and cultured them in osteogenic medium supplemented with Pg-LPS and TNF-α for 7 days. Western blot analysis showed that compared to the inflammatory model group, the protein levels of ALP and RUNX2 were increased in PDLSCs overexpressing miR-21-5p (Figures 7F–H). In order to explore the potential miR-21-5p target genes related to the STAT pathway, we conducted analysis using human public databases (including miRDB, TargetScan, and starBase) and merged them with 88 JAK-STAT genes. The results indicated that STAT3 may be a potential target gene of miR-21-5p (Figure 7I). Western blot analysis confirmed that miR-21-5p significantly inhibited the activation of the STAT3 pathway induced by Pg-LPS and TNF-α in PDLSCs (Figure 7J). These results confirmed that miR-21-5p promotes the osteogenic differentiation of PDLSCs in the inflammatory microenvironment induced by Pg-LPS and TNF-α partly by inhibiting the STAT3 signaling pathway.

## 4 Discussion and conclusion

The microenvironment of periodontitis is primarily composed by dental plaque biofilm and excessive inflammatory cytokines, which disrupt the normal functioning of resident stem cells and impair the natural repair potential of alveolar bone. In our study, we found that the inflammatory microenvironment represented by Pg-LPS and TNF-α significantly inhibited the normal function of PDLSCs. To address this, we developed fusion nanovesicles that combined plant-derived SBNVs and MSC-derived TNFR1-NVs to reshape the anti-inflammatory and regenerative niche through various ways including anti-*P. gingivalis*, inhibition of M1 macrophage polarization, and competitive antagonism of TNF-α. Moreover, these FVs alleviated the inflammatory response of PDLSCs induced by Pg-LPS and TNF-α, restoring their proliferation, migration, and osteogenic differentiation capabilities. Mechanistically, miR-21-5p in FVs promoted osteogenic differentiation by inhibiting the STAT3 signaling pathway. This approach provides a new strategy for regenerating damaged bone tissues under inflammatory conditions.

The research on PDNVs is still in its early stage compared to mammalian cell-derived EVs, with several key issues to be solved, including 1) how to gain and preserve high-quality PDNVs; 2) how to obtain PDNVs with specific biological activities, and so on. Firstly, methods for the isolation, purification, and characterization of PDNVs have not been standardized (Huang et al., 2023). Even slight variations in isolation parameters may result in PDNVs with differing purities and biological activity compositions. Currently, the methods employed for PDNVs isolation primarily are mainly based on ultracentrifugation and density gradient centrifugation, which are time-consuming. Furthermore, due to the unique characteristics of each plant, the selection of plant parts for obtaining PDNVs varies (Ou et al., 2023; Ye et al., 2024; Li et al., 2025), which may lead to the specificity of extraction parameters, further posing a challenge to the standardization of isolation methods. Therefore, there is a necessity for a more systematical discussion on the preparation and characterization techniques of PDNVs within the industry to improve quality control. The second issue involves using a high-throughput drug screening platform to verify the biological activities of PDNVs from different sources. However, the current time-consuming extraction techniques are limiting this process (Huang et al., 2023; Lu et al., 2024). A potential solution is to establish precursor materials that are easier to prepare and can represent the biological activities of PDNVs, using them instead of PDNVs for high-throughput drug screening, which would be a breakthrough in the application of PDNVs. In this study, we validated the potential of SBNVs as antibacterial and anti-inflammatory agents for treating periodontitis. Notably, SBNVs showed functional differences in Pg-LPS inflammation model and TNF-α inflammation model. While Pg-LPS and TNF-α share partial overlap in stimulating inflammatory responses, they activate distinct signaling pathways, which may lead to the functional specificity of SBNVs. Therefore, further studies are needed to elucidate the specific mechanisms of these two inflammation-inducing factors.

Treatment strategies targeting TNF-α may offer new directions for the treatment of periodontitis (Zhang et al., 2024; Ouyang et al., 2025). However, cytokine-targeted therapy for periodontitis is currently limited. In our study, TNFR1-NVs effectively acted as decoy receptors to capture and clear TNF-α *in vitro*. This concept continues the previous research achievements of our research group, but with a significant difference, as this study selects MSCs with immunomodulatory properties as the vector for genetic engineering, which is consistent with the results that TNFR1-NVs eliminate the negative effects of TNF-α through the immune regulatory properties and the engineered design (Xi et al., 2023).In addition to periodontitis, TNFR1-NVs derived from MSCs also hold great potential in the treatment of bone loss characterized by TNF-α, such as osteoarthritis. Furthermore, MSC-derived cell membrane vesicles provide a novel therapeutic approach for targeting cytokine of host immune responses due to their good tissue penetrability, engineerable targeting capability, and low immunogenicity.

Compared to mammalian cell-derived EVs, PDNVs may lack certain specific proteins or glycans on their surface due to their different origin, potentially limiting their tissue-specific targeting (Zhao et al., 2024a). Additionally, there are currently few reports on surface modification methods to improve the tissue targeting ability of PDNVs. Besides, PDNVs are more difficult to undergo genetic engineering for functional modifications limited by existing technology (Bai et al., 2024). To address these problems, early studies by our group have shown that plant-animal fusion nanovesicles have the advantage of improving synergistic therapeutic effects and enhancing targeting (Huang et al., 2023). Therefore, we proposed to fuse SBNVs with engineered PDLSCs-derived NVs. Our results showed that FVs achieved synergistic therapeutic effects, with outcomes superior to each individual component. This synergistic effect may stem from the complementary and comprehensive effect of active ingredients from both sources. In addition, FVs could further enhance the therapeutic effects as drug carriers.

In conclusion, this study creatively developed multifunctional fusion nanovesicle integrating SBNVs and TNFR1 nanovesicles derived from genetically PDLSCs to reshape the anti-inflammatory and regenerative niche. This study represents a nanotherapeutic strategy of utilizing fusion nanovesicles derived from both plant and animal sources as regulators of the inflammatory microenvironment, holds great potential for treating periodontitis.

## Data Availability

The original contributions presented in the study are included in the article/Supplementary Material, further inquiries can be directed to the corresponding authors.
