# Supplementary material for: Plant and animal-derived fusion nanovesicles rescue inflammation-compromised osteogenic potential of periodontal ligament stem cells
Source: Front Cell Dev Biol. 2025 Feb 27;13:1512238. doi: 10.3389/fcell.2025.1512238 (PMC11903722; doi:10.3389/fcell.2025.1512238)
Supplement: Supplementary file 1 [file DataSheet1.docx]

Supplementary Material

Supplementary Table 1. Primers for quantitative real-time PCR.

Supplementary Figure 1. SBNVs have anti-*P. gingivalis* and anti-inflammatory effects. (related to Figure 2).

Supplementary Figure 2. Characterization of PDLSCs. (related to Figure 4).

Supplementary Figure 3. Confocal images showing the uptake of FVs in PDLSCs. (related to Figure 5).

Supplementary Figure 4. Characterization of FVs. (related to Figure 5).

**Supplementary Table 1**. Primers for quantitative real-time PCR.

| Gene | Forward primer sequence | Reverse primer sequence |
| --- | --- | --- |
| Mouse-TNF-α | AAGTCAACCTCCTCTCTGCC | TCCAAAGTAGACCTGCCCG |
| Mouse-IL-6 | TAGTCCTTCCTACCCCAATTTCC | TTGGTCCTTAGCCACTCCTTC |
| Mouse-IL-1β | TGACCTGGGCTGTCCTGATG | GGTGCTCATGTCCTCATCCTG |
| Mouse-β-Actin | GTGACGTTGACATCCGTAAAGA | GCCGGACTCATCGTACTCC |
| Human-IDO1 | TCTCATTTCGTGATGGAGACTGC | GTGTCCCGTTCTTGCATTTGC |
| Human-IL10 | TCAAGGCGCATGTGAACTCC | GATGTCAAACTCACTCATGGCT |
| Human-TNF-α | GGACACCATGAGCACTGAAAGC | TGCCACGATCAGGAAGGAGAAG |
| Human-IL-6 | ACTCACCTCTTCAGAACGAATTG | CCATCTTTGGAAGGTTCAGGTTG |
| Human-IL-1β | ATGATGGCTTATTACAGTGGCAA | GTCGGAGATTCGTAGCTGGA |
| Human-β-Actin | CATGTACGTTGCTATCCAGGC | CTCCTTAATGTCACGCACGAT |
| Human-ALP | CACTATGTCTGGAACCGCACTG | AAGCCTTTGGGATTCTTTGTCA |
| Human-RUNX2 | TGGTTACTGTCATGGCGGGTA | TCTCAGATCGTTGAACCTTGCTA |
| U6 | CTCGCTTCGGCAGCACA | AACGCTTCACGAATTTGCGT |
| has-miR-21-5p | GCGCGTAGCTTATCAGACTGA | AGTGCAGGGTCCGAGGTATT |


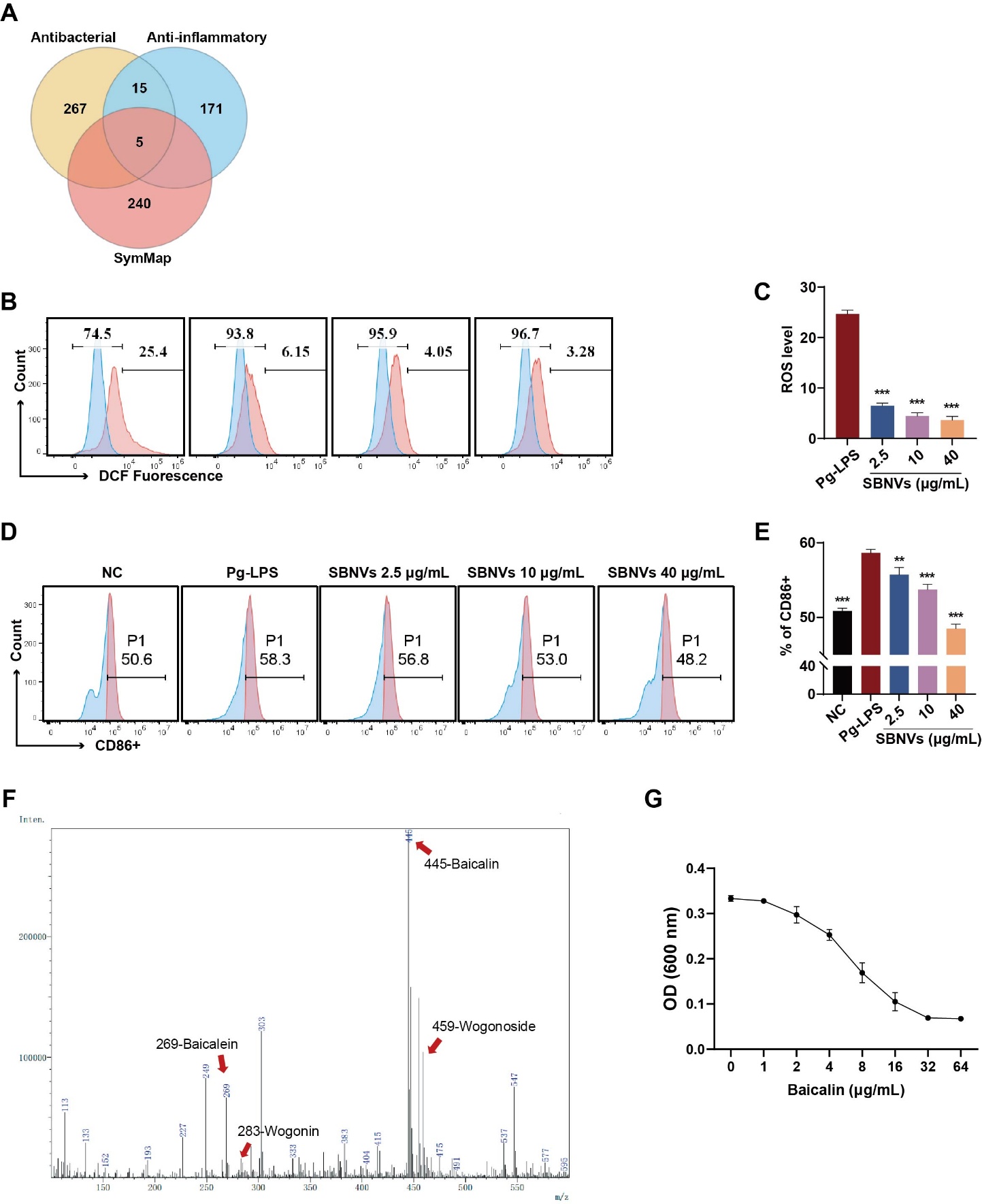


**Supplementary Figure 1.** SBNVs have anti-*P. gingivalis* and anti-inflammatory effects. (a) Bibliometric analysis of plants with anti-*P. gingivalis* and anti-inflammatory effects in the treatment of periodontitis. (b-c) Flow cytometry detection of the ROS levels of Pg-LPS-induced Raw264.7 cells after treatment with different drug groups and the quantitative analysis (n=3). (d-e) Flow cytometry detection of the CD86 positivity rate of Pg-LPS-induced Raw264.7 cells after treatment with different drug groups and the quantitative analysis (n=3). (f) Mass spectrometry analysis of SBNVs in the 100-600 m/z range in negative ion mode. (g) *In vitro* anti-*P. gingivalis* activity of baicalin（n=3）. **p<0.01; ***p<0.001.


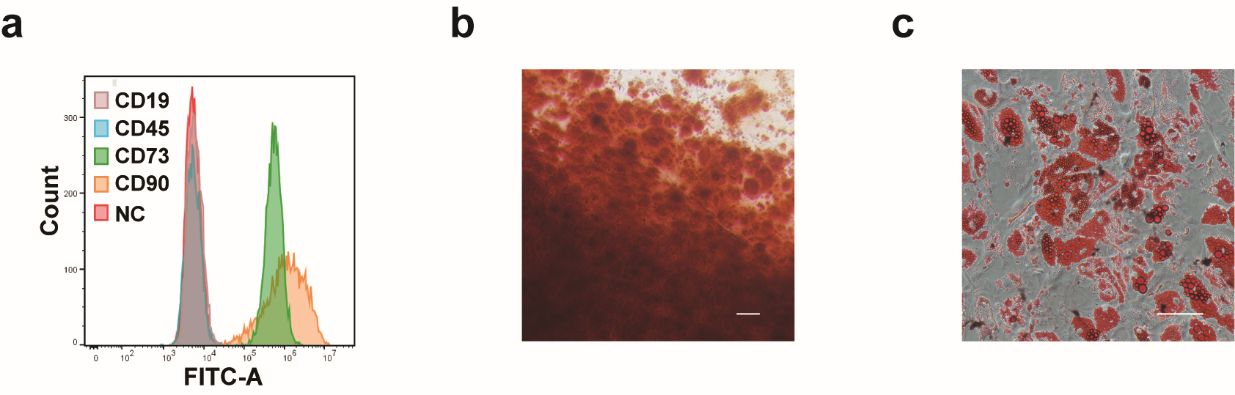


**Supplementary Figure 2.** Characterization of PDLSCs. (a) Identification of MSC markers in P2 generation PDLSCs by flow cytometry. (b-c) Representative images of induced osteogenic and adipogenic differentiation of PDLSCs. Scale bar: 100 μm.


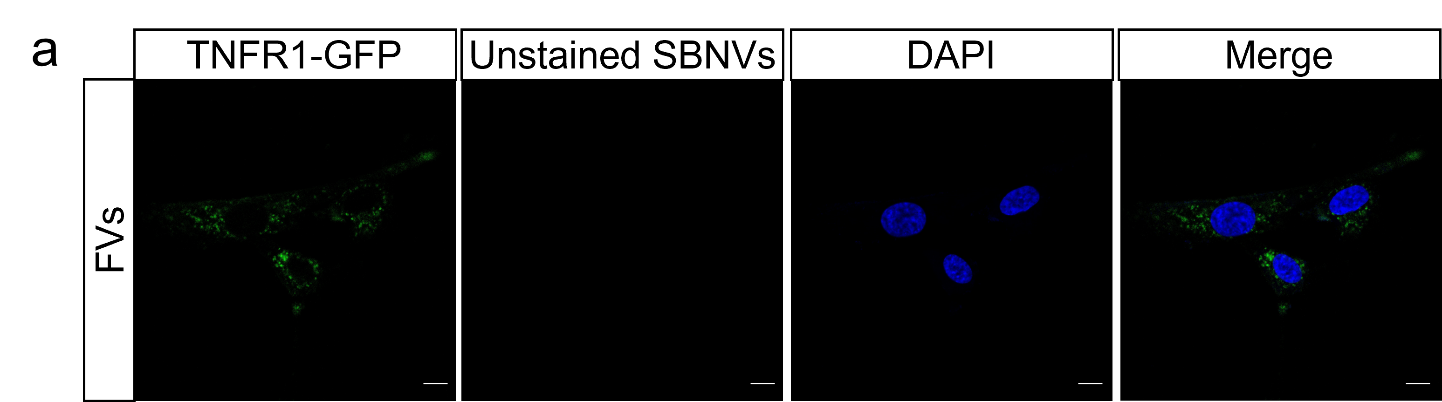


**Supplementary Figure 3**. Confocal images showing the uptake of FVs in PDLSCs. FVs were prepared with unstained SBNVs and TNFR1-NV. DAPI was used to label cell nuclei. Scale bar: 10 μm.


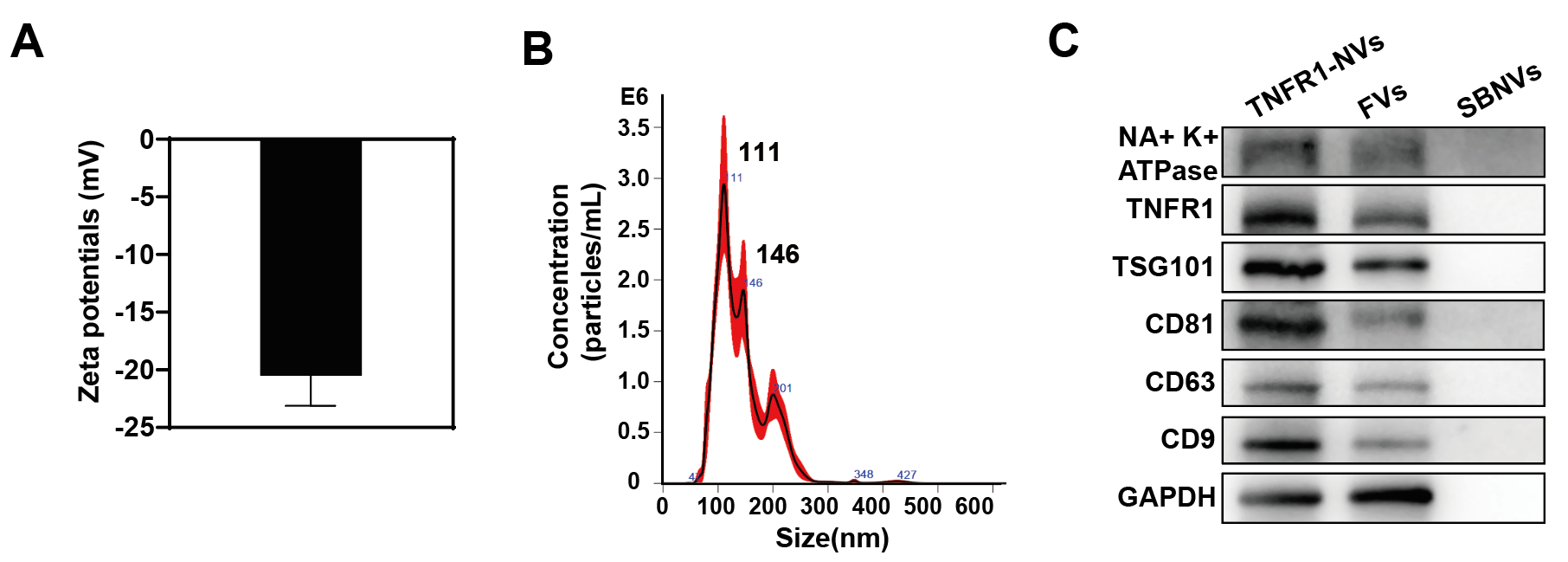


**Supplementary Figure 4**. Characterization of FVs. (a) PALS determination of zeta potential of FVs (n=3). (b) Size distribution and concentration of FVs were determined by NTA. (c) Western blot to detect the expression of related marker proteins Na^+^K^+^ ATPase, TNFR1, TSG101, CD81, CD63 and CD9 in TNFR1-NVs, FVs and SBNVs.
